# Orthodontic Treatment and Craniocervical Posture in Patients with Temporomandibular Disorders: An Observational Study

**DOI:** 10.3390/ijerph18063295

**Published:** 2021-03-23

**Authors:** Maria Paço, José Alberto Duarte, Teresa Pinho

**Affiliations:** 1 CESPU, Instituto de Investigacão e Formação Avançada em Ciências e Tecnologias da Saúde, 4585-116 Gandra-Paredes, Portugal; maria.paco@ipsn.cespu.pt; 2 CIAFEL, Faculdade de Desporto da Universidade do Porto, 4200-450 Porto, Portugal; jarduarte@fade.up.pt; 3 IBMC—Inst. Biologia Molecular e Celular, i3S—Inst. Inovação e Investigação em Saúde, Universidade do Porto, 4585-116 Porto, Portugal

**Keywords:** cephalometry, cervical spine, posture

## Abstract

Orthodontic treatment acts through the application of forces and/or by stimulating and redirecting the functional forces within the craniofacial complex. Considering the interrelationship between craniomandibular and craniocervical systems, this intervention may alter craniocervical posture. Thus, our aim is to (a) compare craniocervical posture, hyoid bone position, and craniofacial morphology before, after, and also in the contention phase at least one year after the orthodontic treatment, in patients with temporomandibular disorders and (b) to verify whether the presence of condylar displacement, the skeletal class, or the facial biotype interferes with the abovementioned outcomes. To do so an observational, analytical, longitudinal, and retrospective design study was carried out. A non-probabilistic convenience sampling method was applied. The sample consisted of clinical records of patients diagnosed with temporomandibular disorders in order to compare pre-orthodontic treatment with post-orthodontic treatment (*n* = 42) and contention phase data (*n* = 26). A cephalometric analysis of several variables was performed. The *p*-value was set as 0.05. When the pre- and post-orthodontic treatment data were analyzed, there were statistically significant changes in variables concerning craniocervical posture (CV angle, C0-C1, and AA-PNS) and also concerning hyoid bone position (C3-Rgn). When pre- and post-orthodontic treatment and contention phase data were analyzed the variables concerning craniocervical posture (C0-C1, CVT/Ver, NSL/OPT, NSL/CVT, NSL/Ver; OPT/CVT, OPT/Ver) and facial biotype had statistically significant changes. This allowed us to conclude that in the sample studied, there were significant differences regarding hyoid bone position (pre- versus post-orthodontic treatment) and craniocervical posture (pre- versus post-orthodontic versus contention), with the craniocervical posture being prone to return to basal values. The presence of condylar displacement was found to significantly increase the H-H1 distance in the three moments of evaluation. Facial biotype was found to significantly increase the NSL/Ver angle on hypodivergent compared to hyperdivergent in the contention phase.

## 1. Introduction

“Although scientific studies do not strongly link orthodontic therapy with the development or prevention of TMD (temporomandibular disorders), it is difficult to imagine a specialty that routinely and significantly changes a patient’s occlusal condition would not have a powerful effect on the masticatory structures and their functions” [1]. This was pointed out by Okeson, and reflects some of the controversies regarding the effects of orthodontic treatment on TMDs [2,3,4,5,6,7,8,9,10,11,12,13]. Though some studies identify small associations between orthodontics and TMDs, they fail to isolate a single unique aspect that can either refute or support this association [3]. One of the possible explanations to these controversial results is the heterogeneity of TMDs, a multifactorial entity without a well-defined etiopathogenesis [14,15,16,17,18,19] that encompasses several conditions, such as temporomandibular joint pain, mastication muscle pain, or a combination of both [15,16,17,20,21]. Having this, when there is a need for orthodontic treatment to target malocclusion [2,3,22,23] concomitant to TMDs, the clinician should be aware of signs and symptoms associated with TMDs and adjust the clinical management before and during the orthodontic treatment [24].

Moreover, the close relationship between the craniomandibular and craniocervical systems has been described, showing their functional, biomechanical, neurodynamic, and physiological interrelationship as both having the potential to influence each other reciprocally [25,26,27,28,29,30,31,32,33,34,35,36,37,38]. Taking this into account, it seems possible that the mechanical effects from orthodontics may lead to muscular and articular adaptations on the cervical spine. Furthermore, considering that a craniocervical dysfunction may act as a contributing factor to TMDs [15,25,26,28,36,37,38,39,40,41,42,43,44,45,46,47,48,49], it is reasonable to assume that the clinician should evaluate any change in the craniocervical region during orthodontic treatment, as well as any change in the TMDs complaints.

Thus, since the relationship between orthodontic treatment and craniocervical posture has not been fully addressed so far, the main objective of this study was to compare craniocervical posture, hyoid bone position, and craniofacial morphology before and after orthodontic treatment and also in the contention phase in patients with TMDs. A secondary objective was to verify whether the presence of condylar displacement, the skeletal class, or the facial biotype interfere with the abovementioned outcomes.

## 2. Materials and Methods 

### 2.1. Study Design

This was an observational, analytical, longitudinal, and retrospective design. A non-probabilistic convenience sampling method was applied, accessing clinical documentation from patients that were submitted to orthodontic treatment and had a clinical diagnose of TMDs. The sample consisted of clinical records of 42 patients treated by the same specialist and PhD in orthodontics (Pinho, T.) to compare pre-orthodontic treatment to post-orthodontic treatment. From this initial sample a sub-group of 26 clinical records (that contained a teleradiograph from one year after orthodontic treatment) was analyzed in order to compare pre- and post-orthodontic treatment and contention phase data.

To be included in the study the patients had to be examined for clinical history (clinical diagnosis of TMDs, based on Diagnostic Criteria for Temporomandibular Disorders—DC/TMD), lateral and anterior photographs (in a natural head position), have good-quality teleradiography (also in natural head position and should include the head and cervical column, with at least the fourth cervical vertebra completely visible), have dental casts mounted on an articulator in a centric relation, and be aged at the beginning of orthodontic treatment between 18 and 50 years old. Another inclusion criterion was the achievement of a canine Class I relation and normalized overjet and overbite values after orthodontic treatment. 

Cases were excluded if they presented history of traumatic injuries, fibromyalgia syndrome, diagnosis of systemic disease, or presence of neurological disorders. 

Ethical approval was guaranteed by the Ethics Committee at Instituto Universitário de Ciências da Saúde, CESPU (3/CE-IUCS/2016).

### 2.2. Procedures

After checking the eligibility of the cases, the assessment of craniocervical posture, hyoid bone position, craniofacial morphology, and occlusal factors was performed. 

The occlusal parameter studied was the presence of malocclusions and condylar displacement, using intra-oral photographs as well as dental casts. Furthermore, the mounting models were adopted in a centric relation on a semi-adjustable articulator SAM 3^®^ (Präzisionstechnik, Taxisstr. 41, München, Germany) and the register of the condyle position and consequently the amount of condylar displacement was registered with a mandible position indicator (MPI 120^®^, Präzisionstechnik, Taxisstr. 41, München, Germany). These procedures were previously described and considered reliable [50,51,52]. When the condylar displacement was analyzed, it was considered that a Δ ≥ 2 mm was consistent with a higher risk of developing TMDs, and the participants were classified as “condylar displacement present” [23,53,54,55,56,57]. 

Regarding the craniocervical posture, hyoid bone position, and craniofacial morphology analyses, these were performed by teleradiograph cephalometric analysis with lateral photograph superimposition (both in a natural head position) with Nemoceph^®^ software (Nemoceph 6—Dental Studio NX, version 6.0, Madrid, Spain)^®^. The method for obtaining the lateral cephalogram in a natural head position was performed as previously described [58] and the lateral photographs (also in a natural head position) used for superimposition allowed for the confirmation of a natural head position.

A natural head position was obtained following the procedures established in the literature. The cephalometric points used were marked as previously described [59,60,61,62,63,64] and are defined in Table 1. 

Lateral cephalograms of 10 randomly selected subjects were measured twice, with a one-week interval between measurements, to assess the magnitude of measurement errors (intraclass correlation coefficient (ICC)_(2,1)_). The ICC_(2,1)_ for the reliability of the landmark identification was 0.98, demonstrating an excellent reliability [65]. 

### 2.3. Statistical Analysis

Statistical analysis was performed using the Statistical Package for the Social Sciences (SPSS)^®^, version 24 (IBM, Chicago, IL, USA). To assess the normal distribution of the variables, the Shapiro–Wilk test was applied. Sample characteristics are presented as absolute frequencies in categorical variables and mean and standard deviation (SD) in quantitative variables. The presence of potential differences between pre- and post-intervention results were analyzed through a paired samples *t*-test or Wilcoxon test, respectively, for whether the outcomes had a normal distribution or not. A repeated-measures ANOVA was used to evaluate the presence of potential differences between the three assessment moments (pre-intervention, post-intervention, and contention phase). The assumptions to perform this test were normal distribution of the variables (Shapiro–Wilk test) and sphericity (Mauchly’s test). When the sphericity assumption was not fulfilled, the F-value was corrected, in accordance with previously described methods [66]. Multiple comparisons between the three assessment moments were performed through a Bonferroni post-hoc test. When the assumptions for parametric tests were not fulfilled, the Friedman test was used, and multiple comparisons were performed through Wilcoxon tests. To compare the outcome variables, according to the presence or absence of condylar displacement, independent samples *t*-tests or Mann–Whitney tests were used as parametric and non-parametric tests, respectively. To compare the outcome variables according to the skeletal class and facial biotype, the one-way ANOVA (with a Bonferroni post-hoc test) and Kruskal–Wallis tests were used as parametric and non-parametric tests, respectively. The critical value for significance in all the analysis was *p*-value < 0.05.

## 3. Results

The sample regarding pre and post orthodontic treatment results consisted of 42 individuals (6 men, 36 women), aged 28.14 ± 11.36 years in the beginning of the treatment and the duration of orthodontic treatment was 2.87 ± 1.45 years.

Data regarding facial and skeletal characteristics of the participants and pre-orthodontic treatment are presented in Table 2.

Table 3 presents the variables that had statistically significant changes when comparing the values for pre-orthodontic treatment to the values for post-orthodontic treatment.

When the cephalometric variables were adjusted to the presence or absence of condylar displacement, to the skeletal class, and also to the facial biotype, there were no significant differences among the different groups in any of the assessment moments, except for the variable H-Rgn, with differences between skeletal Class I (43.69 ± 4.33) and Class II (39.72 ± 5.55) after orthodontic treatment (*p* = 0.009).

When the subgroup of participants with data regarding the contention phase was analyzed, the total number of participants was 26 (4 men, 22 women), aged 27.77 ± 8.49 years old at the beginning of the treatment.

Table 4 presents the variables that had statistically significant changes, when comparing pre-orthodontic treatment to post-orthodontic treatment and tothe contention phase.

When the cephalometric variables were adjusted to the presence or absence of condylar displacement, to the skeletal class, and also to the facial biotype, there were no differences among the different groups in any of the assessment moments, except for the variables H_H1, facial proportion, and NSL/Ver. H_H1 was found to have statistically significant changes between the participants with condylar displacement and those without it before orthodontic treatment (“condylar displacement present” 8.41 ± 3.80; “condylar displacement absent” 2.62 ± 6.24; *p* = 0.031), after orthodontic treatment (“condylar displacement present” 7.63 ± 2.97; “condylar displacement absent” 2.14 ± 7.10; *p* = 0.11), and in the contention phase (“condylar displacement present” 8.16 ± 5.57;“condylar displacement absent” 1.28 ± 6.66; *p* = 0.023).

NSL/Ver was found to have statistically significant changes between hyperdivergent (74.81 ± 3.59) and hypodivergent facial type participants (82.00 ± 2.72) only in the contention phase (*p* = 0.005).

## 4. Discussion

In our study we evaluated the craniocervical posture in participants with a TMDs diagnose who underwent orthodontic treatment. Our participants were mostly hyperdivergent, with a skeletal Class II and a facial proportion that showed an increase in the inferior facial height and a decrease in the submandibular distance. The values of the facial proportion (>1.20) showed a tendency toward Class II with posterior mandible rotation/retrusion, which is indicative of a weak musculature [61].

Our study showed that, after orthodontic treatment, the participants presented an increase in CV angle concomitantly with an increase in C0-C1 distance and in C3-Rgn distance, as well as a decrease in AA-PNS distance. An increase in CV angle is associated with an anterior rotation of the head [67]. This rotation of the head was also corroborated by the decrease in AA-PNS distance, which is usually associated with a flexed craniocervical posture. This finding was also confirmed by the results of the distance of C0-C1, the increase in which reflects the rectification of the cervical column. The increase in the C3-Rgn distance is also compatible with a loss of cervical lordosis. In spite of the variables NSL/OPT and NSL/CVT not presenting statistically significant changes, they did present relevant mean increases, which is also compatible with an anterior rotation of the head.

After adjustment of the cephalometric variables according to the presence or absence of condylar displacement, the skeletal class, and the facial biotype, the results showed that the presence of condylar displacement was found to significantly increase the H-H1 distance at the three moments of evaluation compared to the participants without condylar displacement. This distance increase is associated with a downward position of the hyoid bone and may reflect muscular asymmetry between supra and infra-hyoid muscles. Facial biotype was found to significantly increase the NSL/Ver angle in hypodivergent compared to hyperdivergent participants in the contention phase. This result is compatible with a posterior rotation of the head and a forward inclination of the cervical column, which seems to be related to a hyperdivergent morphology and retrognathic profile.

This study also intended to assess the stability of the results obtained and did so by evaluating the presence of TMD signs and/or symptoms, the craniocervical posture, hyoid bone position, and craniofacial morphology (including dental class and overbite and overjet values) in the contention phase (one year after finishing orthodontic treatment) and comparing to pre-orthodontic treatment and post-orthodontic treatment data. This comparison was performed in a subgroup of the initial sample. When the results obtained were analyzed, all the patients remained TMD sign and symptom free, had no relapse in dental class, and overbite and overjet values remained within normal values.

On the other hand, significant changes were found mainly in the craniocervical posture variables and also in the facial biotype, which demonstrated a tendency toward normodivergence. The craniocervical posture variables that had statistically significant changes (C0-C1, CVT/Ver, NSL/OPT, NSL/CVT, NSL/Ver, OPT/CVT, OPT/Ver) had differences compatible with a posterior rotation of the head and an extended cervical column that highlights an increase in cervical lordosis, which is thought to increase the load on the posterior cervical structures [35], where an excessive capsular ligament stretch, beyond the biophysical limitations, could decrease the threshold of nerve endings and activate proprioceptors in facet joint capsules, which have a role in the development of cervical muscle pain [68]. These differences had a particular impact when “pre-orthodontic treatment” versus “contention phase” and “post orthodontic treatment” versus “contention phase” were analyzed. It was interesting to observe that in the majority of the measures that had significant changes (NSL/OPT, NSL/CVT, OPT/CVT, OPT/Ver), when “pre orthodontic treatment” versus “post orthodontic treatment” were compared the tendency shown was the opposite (anterior rotation of the head and rectification of the cervical column, although without statistically significant differences).

Keeping in mind the results found for the interrelationship between craniomandibular and craniocervical systems and considering the fact that the literature showed the shared pathophysiological mechanisms between TMDs and cervical spine disorders with craniocervical dysfunction having the potential to lead to or perpetuate TMDs [42,43,44,45,46,47,48], it is conceivable that craniocervical changes have the potential to contribute to the occlusal and/or TMD symptom relapse seen in clinical practice and described in the literature [69,70].

The reduced sample size was the result of our inclusion criterion regarding the presence of TMDs. This fact allowed us to be more specific regarding TMD sufferers; however, it narrowed the sample that we had access to. Furthermore, it did not allow us to analyze the data according to TMD classification, which could have interfered with our results interpretation. Different types of TMDs have different clinical characteristics and may have interfered with the outcomes studied. However, despite the sample size, the effect sizes are important. Another factor to bear in mind is that the diagnosis of TMDs was a clinical diagnosis based on DC/TMD. This choice was due to the fact that during data collection the DC/TMD was not available in Portuguese (Portugal). It is also important to highlight the controversy regarding orthodontics and TMDs, with studies reporting good results on TMDs resolution or, at least, on reducing the risk of patients developing it, whereas other studies suggest that orthodontic treatment increases the risk of onset of signs and symptoms of TMDs or is TMD neutral [2,4,5,6,7,8,9,10,11]. In our study, and due to its methodological design, we did not aim to establish any causal relation between orthodontics and TMDs nor craniocervical posture, but rather to characterize the craniocervical posture before and after orthodontic treatment and also in the contention phase in patients with TMDs. Another factor to take into account is the evaluation of head and neck posture, which was performed in a resting position (natural head position). This is the position often used not only in research but also in clinical settings. However, considering that all individuals assume many different head and neck postures throughout their daily tasks, future studies should account for the dynamics of the cervical spine instead of focusing on rest positions, as suggested by Kraus [71].

Regarding the orthodontic treatment plan of our sample, it was performed taking into account the dental problems as well as the TMD condition. Although it has been established that orthodontic therapy neither causes nor prevents TMDs [11], this therapeutic intervention provides dental and orthopedic stability in the masticatory structures, which will most likely reduce the patient’s risk factors for developing TMDs [72]. Concerning occlusal changes, mispositioned teeth associated with some malocclusions with steeper occlusal planes, posterior prematuries, and mandibular lateral displacement are often associated with craniomandibular dysfunction [73,74,75]. Having this, before planning orthodontic treatment, it is imperative to identify the location of an asymmetry, as treatment protocols will vary depending on the underlying etiology [76,77]. A midline discrepancy is one of the symptoms of mandibular lateral displacement and the major objective of orthodontic correction should be the elimination of any posterior discrepancy and the differential control of the occlusal plane. Therefore, by increasing the occlusal vertical height of the shifted or affected side, balanced muscle and articular disc positions can be restored, which is expected to contribute to the patient’s TMD symptom resolution [74]. Furthermore, orthodontic treatment is aimed at controlling the occlusal plane, with consequent improvement of occlusal and articular functions [73,75,78]. Since occlusal factors may be a potential source of TMDs, improving occlusion will certainly minimize any risk factors that might be associated with TMDs [72].

In the present study, in the cases where the patients remained with a clinical diagnosis of TMDs after orthodontic treatment (*n* = 3; 7.14%), they were further evaluated by a specialist in orofacial pain who provided the treatment needed to specifically address this clinical condition. 

It is also important to highlight the fact that, in our sample, six participants (14,29%) had a loss of posterior teeth and, in these cases, the major objective of orthodontic correction was the elimination of any posterior discrepancy and the differential control of the occlusal plane [73]. The reader should bear in mind this fact, since this could potentially affect the facial morphology and the head and neck posture.

Because there are no standardized values for most of the variables studied, we did not intend to classify the final result as normal or abnormal alterations, but mostly to characterize and verify whether there were changes after orthodontic treatment and in the contention phase. The presence of changes was interpreted as a signal of the interrelationship between craniomandibular and craniocervical systems, alerting the clinician to the necessity of addressing these alterations during the treatment and contention phase, since they may contribute to the development/aggravation of TMDs signs and/or symptoms, and also acknowledging that during orthodontic treatment other factors that interfere with general health status and quality of life may change. This monitoring is important since several studies have shown that an altered craniocervical posture seems to influence the process of myofascial pain sensitization in the cervical muscles, which could lead to the development of the referenced pain in the masticatory muscles [46,63,79,80]. Moreover, a recent systematic review and meta-analysis found that there was a significant correlation with a moderate clinical effect between neck disability and jaw disability in patients with TMDs [40]. In addition, a recent review by Gil-Martínez et al. [39] reported that neck disability was a strong predictor of craniofacial pain and disability in a subgroup of patients with TMDs due to muscle pain and that neck disability has a positive correlation with orofacial pain and disability, kinesiophobia, and pain catastrophizing. 

In a clinical perspective, this knowledge about postural adaptations during orthodontic treatment should create awareness in the medical community, highlighting possible impairments that should be evaluated in order to achieve the greatest results for the patient.

Thus, it seems important to conduct well-designed longitudinal and randomized controlled trials comparing craniocervical posture as well as TMD signs and symptoms before and after the orthodontic treatment and a follow-up period superior to the contention phase (one year) in individuals diagnosed with TMDs, stratified according to TMD classification system.

## 5. Conclusions

Our results demonstrated that in the sample studied there were statistically significant differences regarding hyoid bone position (pre orthodontic treatment versus post orthodontic treatment) and craniocervical posture (between the three moments of evaluation: pre-orthodontic treatment, post-orthodontic treatment, and contention phase), with the craniocervical posture being prone to return to basal values. The presence of condylar displacement was found to significantly increase the H-H1 distance in the three moments of evaluation. Facial biotype was found to significantly increase the NSL/Ver angle in hypodivergent compared to hyperdivergent participants in the contention phase.

## Figures and Tables

**Table 1:** Cephalometric landmarks, angles, and reference measures.

Measure	Definition
Craniovertebral angle (CV angle)	The angle resulting from the intersection between a horizontal line that goes from the Bolton point (Bo) (the intersection of the outline of the occipital condyle and the foramen magnum at the highest point on the notch posterior to the occipital condyle) to the posterior nasal spine and the vertice of the odontoid process and the anteroinferior point of the odontoid process.
C0-C1	The distance between the horizontal line that goes from the posterior nasal spine and the most anterior point of the first cervical vertebra.
C1-C2	The distance between the most anterior aspect of the first cervical vertebra and the second cervical vertebra.
C3-H	The distance between the most anterior aspect of the third cervical vertebra and the most anterior point of the hyoid bone.
C3-Rgn	The distance between the most anterior aspect of the third cervical vertebra and the most dorsal and inferior point of mandibular symphysis (retrognation).
H-H1	The distance from the most anterior point of the hyoid bone and the horizontal line that goes from the most anterior aspect of the third cervical vertebra and retrognation.
H-Rgn	The distance from the most anterior point of the hyoid bone and the retrognation.
AA-PNS	The distance from the most anterior point of the atlas vertebra (AA) to the posterior nasal spine.
CVT/Ver	The angle resulting from the intersection between the tangent that goes posterior to the odontoid process through the most posterior and inferior aspect of the fourth cervical vertebra body and the vertical line that corresponds to the true vertical.
NSL/CVT	The angle resulting from the intersection between a line that goes from the sela turcica to the nasion and the tangent that goes posterior to the odontoid process through the most posterior and inferior aspect of the fourth cervical vertebra body.
NSL/OPT	The angle resulting from the intersection between a line that goes from the sela turcica to the nasion and the tangent that goes posterior to the odontoid process through the most posterior and inferior aspect of the second cervical vertebra body.
NSL/Ver	The angle resultant from the intersection between a line that goes from the sela turcica to the nasion and the vertical line that corresponds to the true vertical.
OPT/CVT	The angle resulting from the tangent that goes posterior to the odontoid process through the most posterior and inferior aspect of the second cervical vertebra body and the tangent that goes posterior to the odontoid process through the most posterior and inferior aspect of the fourth cervical vertebra body.
OPT/Ver	The angle resulting from the intersection between the tangent that goes posterior to the odontoid process through the most posterior and inferior aspect of the second cervical vertebra body and the vertical line that corresponds to the true vertical.
Facial biotype	Through the measurement of FMA, where a score less than 22 means hypodivergent, between 22 and 28 means normodivergent, and higher than 28 means hyperdivergent.
Skeletal class	Through the measurement of ANB, where a score inferior to 0 represents Class III, between 0–5 represents Class I, and a score superior to 5 represents Class II.
Facial proportion	Calculated by the intersection ratio of the Sn-Gnc line with the Gnc-C line.

**Table 2:** Sample characterization regarding skeletal class, facial biotype, and condylar displacement before orthodontic treatment (*n* = 42).

Characteristics		Frequency (%)
Skeletal Class	Skeletal Class I	45.2
	Skeletal Class II	50
	Skeletal Class III	4.8
Facial Biotype	Hypodivergent	16.7
	Normodivergent	23.8
	Hyperdivergent	59.5
Condylar Displacement	Present	23.8
	Absent	76.2

**Table 3:** Cephalometric variables at two moments: pre-orthodontic treatment and post-orthodontic treatment (*n* = 42).

Cephalometric Variable	Pre OT Mean (SD)	Post OT Mean (SD)	*p*-Value (Paired Samples *t*-Test)
Craniocervical Posture			
CV angle	99.90 (11.65) *	98.10 (13.00) *	0.036 ^†^
C0-C1	6.75 (4.01)	7.84 (3.96)	0.017
C1-C2	20.15 (2.18)	20.80 (2.35)	NS
CVT/Ver	7.42 (7.32)	7.38 (8.07)	NS
NSL/OPT	78.50 (15.25) *	78.30 (9.30) *	NS†
NSL/CVT	92.94 (7.45)	95.34 (8.22)	NS
NSL/Ver	79.67 (4.30)	77.26 (4.49)	NS
OPT/CVT	15.72 (4.80)	15.10 (4.54)	NS
OPT/Ver	23.14 (9.21)	22.48 (10.64)	NS
AA-PNS	36.53 (4.35)	35.61 (4.41)	0.009
Hyioid Bone Position			
C3-H	36.60 (3.92)	36.98 (4.36)	NS
C3-Rgn	74.70 (8.49)	76.80 (7.84)	0.018
H-H1	5.11 (6.14)	4.31 (6.04)	NS
H-Rgn	40.15 (6.46)	41.26 (5.42)	NS
Craniofacial Morphology			
Facial biotype	28.68 (7.10)	29.02 (7.12)	NS
Skeletal class	4.88 (3.03)	5.11 (3.02)	NS
Facial proportion	1.50 (0.30)	1.46 (0.28)	NS

* Median (interquartile range); ^†^ Wilcoxon test; SD—standard deviation; OT—orthodontic treatment; NS—non-significant.

**Table 4:** Cephalometric variables at three moments: pre-orthodontic treatment (Pre-OT), post-orthodontic treatment (Post-OT), and contention (*n* = 26), and respective *p*-values.

Cephalometric Variable	Pre-OT Mean (SD)	Post-OT Mean (SD)	Contention Mean (SD)	*p*-Value (ANOVA Repeated Measures)	Multiple Comparisons *p*-Value (Bonferroni)
Craniocervical Posture					
CV angle	98.99 (8.92)	97.72 (9.60)	96.87 (8.99)	NS	-
C0-C1	8.50 (6.00) *	9.40 (5.50) *	9.60 (4.45) *	0.028 ^†^	0.002 ^‡^ (Pre-OT/Contention)
C1-C2	19.96 (2.35)	20.64 (2.39)	21.14 (2.79)	NS	0.033 (Pre-OT/Contention) <0.001 (Post-OT/Contention)
CVT/Ver	7.20 (12.05) *	9.00 (13.20) *	13.90 (12.05) *	<0.001 ^†^	<0.001 ^‡^ (Pre-OT/Contention) <0.001 ^‡^ (Post-OT/Contention)
NSL/OPT	75.00 (18.70) *	77.90 (11.60) *	68.40 (16.45) *	<0.001 ^†^	0.033 ^‡^ (Pre-OT/Contention) <0.001 ^‡^ (Post-OT/Contention)
NSL/CVT	93.33 (7.84)	95.44 (9.88)	88.71 (9.70)	<0.001	0.008 (Pre-OT/Contention) <0.001 (Post-OT/Contention)
NSL/Ver	79.18 (3.81)	76.90 (4.10)	75.90 (4.38)	<0.001	0.008 (Pre-OT/Contention) <0.001 (Post-OT/Contention)
OPT/CVT	15.24 (6.44)	14.86 (5.11)	17.97 (4.90)	0.011	0.027 (Post-OT/Contention)
OPT/Ver	22.74 (10.51)	22.52 (11.98)	33.37 (9.51)	<0.001	0.001 (Pre-OT/Contention) <0.001 (Post-OT/Contention)
AA-PNS	37.88 (4.22)	37.17 (4.09)	37.55 (4.20)	NS	-
Hyoid Bone Position					
C3-H	36.70 (4.07)	37.31 (4.67)	37.50 (4.27)	NS	-
C3-Rgn	75.33 (8.38)	77.36 (7.85)	76.70 (6.55)	NS	-
H-H1	3.99 (6.25)	3.32 (6.64)	2.80 (6.93)	NS	-
H-Rgn	40.11 (6.67)	41.40 (5.47)	40.66 (5.23)	NS	-
Craniofacial Morphology					
Facial biotype	29.54 (7.34)	29.75 (6.11)	29.10 (7.81)	<0.001	0.008 (Pre-OT/Contention) <0.001 (Post-OT/Contention)
Skeletal class	5.10 (3.95) *	5.20 (2.95) *	5.30 (3.90) *	NS ^†^	-
Facial proportion	1.46 (0.31) *	1.48 (0.37) *	1.49 (0.36) *	NS ^†^	-

* Median (interquartile range); ^†^ Friedman test; ^‡^ Wilcoxon test; SD—standard deviation; NS—non-significant.
